# Commentary: Oxytocin Enables Maternal Behavior by Balancing Cortical Inhibition

**DOI:** 10.3389/fnbeh.2015.00311

**Published:** 2015-11-18

**Authors:** Ti-Fei Yuan, Gonglin Hou

**Affiliations:** ^1^School of Psychology, Nanjing Normal UniversityNanjing, China; ^2^Department of Psychology, Zhejiang Sci-Tech UniversityNanjing, China

**Keywords:** oxytocin, maternal behavior, brain asymmetry, cortex, inhibition

Oxytocin is a key molecule in social behavior. A recent study reported lateralized oxytocin signaling specifically in left auditory cortex during pup-retrieval behavior of female mice.

Brain asymmetry shows great advantage in evolutionary adaption to the environment (Concha et al., 2012). Left and right (L-R) hemispheres exhibit differences in both brain structure (e.g., volume, surface areas, length interhemispheric, and neuronal density) and the function. In human, the left hemisphere is the dominant hemisphere for verbal cognitive function, while the right hemisphere is more dominant for the spatial cognitive function and mood regulation (Hou et al., 2013). Similar brain asymmetry has also been documented in the visual system and hippocampus from birds and rodents (Halpern et al., 2005). The molecular mechanism underlying the functional asymmetry in physiology and pathology is just begun to be understood (Hou et al., 2015). For example, Kawakami et al. firstly identified the lateralized distribution of NMDA receptor in hippocampus neurons (Kawakami et al., 2003), which might explain the differences in L-R hippocampal cognition.

How are maternal behaviors encoded in the brain? Oxytocin has been known to instruct the parental behavior since 1980s (Pedersen and Prange, 1979; Van Leengoed et al., 1987; Rilling and Young, 2014). Administration of oxytocin could initiate and trigger the maternal behavior, which endures as long-term behavior plasticity. In addition, oxytocin was found to be important for social behavior, trust, reducing fear and stress in brain functions (Lee et al., 2009). Oxytocin signaling in amygdala and nucleus accumbens-ventral pallidum pathways was critical in social bond formation (e.g., pair bond, parental behavior, Numan and Young, 2015). However, it is unclear if the oxytocin signaling and maternal behaviors are encoded symmetrically in the brain. Combing optogenetics, *in vivo* patch clamp configuration, and pup-retrieval behavior paradigm, Marlin et al. set out to fill in this gap (Marlin et al., 2015).

Marlin et al. firstly established a pup retrieval behavior paradigm in mouse. They found that when co-housed with dams, virgin female mice learned to retrieve isolated pups in the cage within 3 days. Interestingly, the oxytocin infusion or optogenetic activation of oxytocin neurons in the paraventricular nucleus (PVN) significantly facilitated the process (1 day). In single housed virgin female mice, oxytocin signaling was sufficient to induce the maternal behavior. Given the important roles of pup calls in triggering the maternal behavior, Marlin et al. examined the expression of oxytocin receptor (OXTR) in the auditory cortex. They then identified large population of OXTR expressing interneurons, suggesting for a potential role of oxytocin in disinhibiting the auditory cortex (Marlin et al., 2015) (Figure 1).

**Figure 1:**
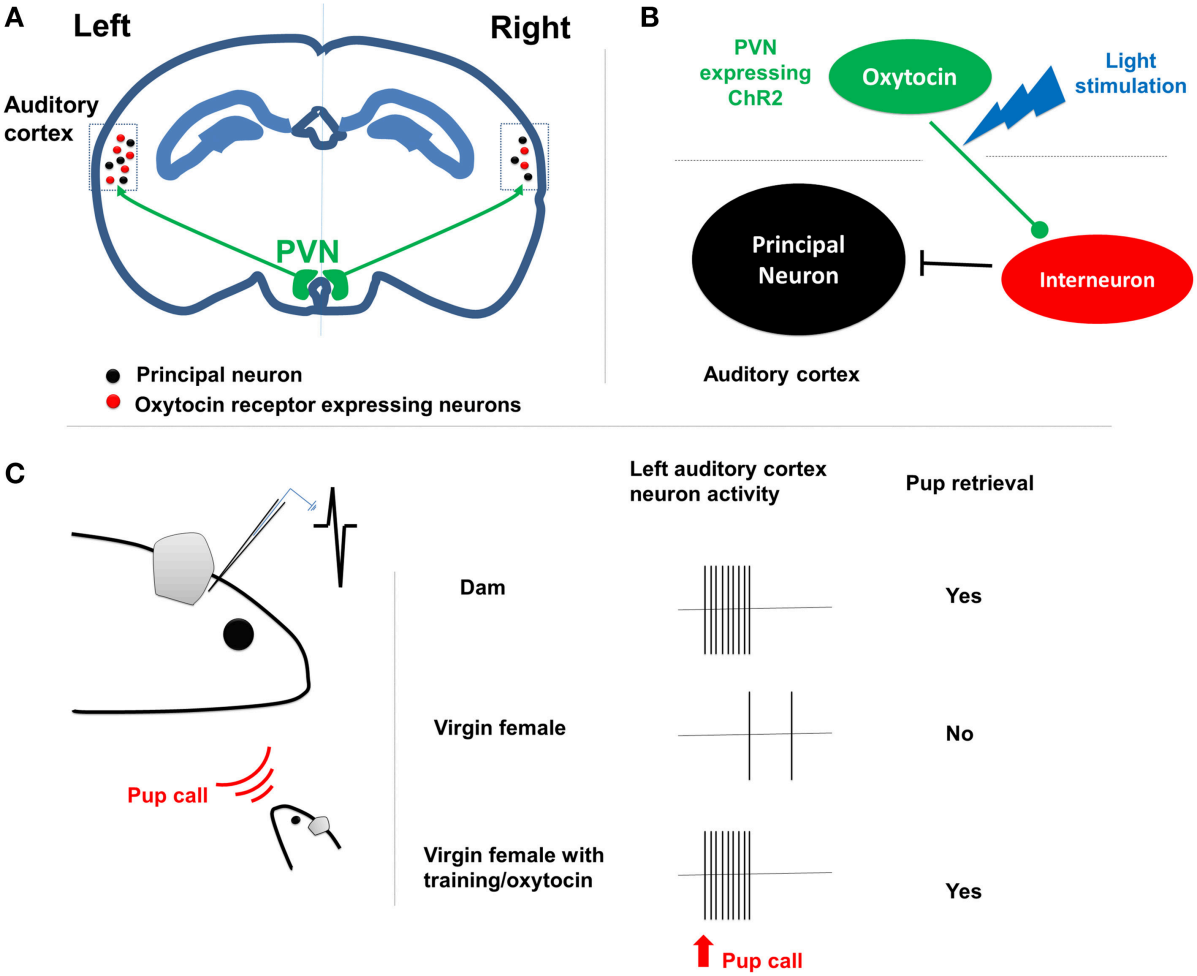
**The lateralization of maternal behavior by oxytocin signaling**. **(A)** PVN oxytocin neurons project to bilateral auditory cortex. In left auditory cortex there are more oxytocin receptor expressing neurons, including many interneurons. **(B)** The oxytocin release from axon terminals shut down the interneurons and disinhibits the cortical principal neurons. **(C)** Left shows the paradigm of *in vivo* patch clamp recording from the left auditory cortex of female dam/virgin mouse when pup call cue is given. Right shows that dam rather than female virgin respond to the pup call both at physiological and behavioral level. The retrieval training experience tuned the cortical neurons to be responsive to pup calls as well.

Indeed, with *in vivo* patch clamp recording, Marlin et al. found reliable activation of auditory cortical principal neurons in dams upon pup call, but not in virgin females. The excitatory-inhibition inputs were well balanced on auditory cortical neurons in dams and virgin female with retrieval experience, but not in the naïve virgin female. Such differences were eliminated either by oxytocin administration or ontogenetically triggered oxytocin release (Marlin et al., 2015). This result suggested that oxytocin orchestrates the maternal behavior by regulating excitatory-inhibition balance, leading to long-lasting plasticity in the auditory cortex, since the oxytocin is not required for the maintenance of the maternal behavior.

Interestingly, Marlin et al. reported clear L-R differences in auditory cortex functioning during the maternal behavior (Marlin et al., 2015). Histological analyses revealed significantly higher number of OXTR expressing neurons in the left auditory cortex in both dams and virgin females, in comparison to the right side. At the functional level, silencing the left but not right auditory cortex with muscimol impaired pup retrieval performance in experienced animals. Furthermore, the oxytocin infusion or optogenetic stimulation of oxytocin fibers selectively at left auditory cortex was sufficient to facilitate the acquisition of the pup retrieval behavior.

It is known that in human subjects, mothers in a harmonious diadic relationship with their children showed greater activation of left hippocampus to child's cry, in compared to those with sensitive/instructive relationship to their children (Musser et al., 2012); in addition, left but not right amygdala is found to be important in differentiating own child's cry (Wan et al., 2014). Such results might indicate a left-hemisphere dominant role in auditory cue induced-maternal behavior in human as well. This is consistent with the fact that verbal cognitive function is important in mother-infant interaction. On the other hand, the visual cues (e.g., images of own child) and mother-infant interactions (e.g., playing) have been shown to activate other brain regions in the mother (Parsons et al., 2010), and these might demonstrate a different lateralization in compared to the auditory cue ones.

Why is oxytocin signaling lateralized to the left hemisphere? It is known that in human social emotion process is dominated by left brain while primary emotions (such as fear) is dominated in the right brain (Ross et al., 1994). Segregation of emotion processing therefore allows rapid identification and response to different types of cues, which are necessary for progeny protection and escape from prey, respectively, for instance. It will be interesting to identify the potential asymmetry of other neural circuits involved in parental behavior, such as medial preoptic area (MPOA), nucleus accumbens-ventral pallidum, and ventral tegmental area (VTA; Stolzenberg and Numan, 2011; Rilling and Young, 2014).

Knowing that oxytocin signaling lateralization is important for maternal behavior raises a new set of questions. What determines the asymmetrical distribution of oxytocin receptor expression between left and right cortical areas? Which cortical area is crucial for each types of maternal behavior and how does oxytocin facilitate the learning process? What other signaling molecules or neural circuits might the brain use for maternal behavior in parallel? How are these maternal cues encoded in infant's brain? The answers to these questions will lead to the further understanding of the most generous love—mother love.

## Author contributions

TY and GH designed the study and wrote the paper together.

### Conflict of interest statement

The authors declare that the research was conducted in the absence of any commercial or financial relationships that could be construed as a potential conflict of interest.
